# High Rate of A(H1N1)pdm09 Infections among Rural Thai Villagers, 2009–2010

**DOI:** 10.1371/journal.pone.0106751

**Published:** 2014-09-04

**Authors:** Benjawan Khuntirat, In-Kyu Yoon, Malinee Chittaganpitch, Whitney S. Krueger, Krongkaew Supawat, Patrick J. Blair, Shannon D. Putnam, Robert V. Gibbons, Darunee Buddhari, Pathom Sawanpanyalert, Gary L. Heil, John A. Friary, Gregory C. Gray

**Affiliations:** 1 Department of Virology, Armed Forces Research Institute of Medical Sciences, Bangkok, Thailand; 2 National Institute of Health, Ministry of Public Health, Nonthaburi, Thailand; 3 College of Public Health and Health Professions and Emerging Pathogens Institute, University of Florida, Gainesville, Florida, United States of America; 4 Naval Medical Research Center - Asia, Singapore, Singapore; 5 Naval Health Research Center, San Diego, California, United States of America; Imperial College London, United Kingdom

## Abstract

**Background:**

Pandemic influenza A(H1N1)pdm09 emerged in Thailand in 2009. A prospective longitudinal adult cohort and household transmission study of influenza-like illness (ILI) was ongoing in rural Thailand at the time of emergence. Symptomatic and subclinical A(H1N1)pdm09 infection rates in the cohort and among household members were evaluated.

**Methods:**

A cohort of 800 Thai adults underwent active community-based surveillance for ILI from 2008–2010. Acute respiratory samples from ILI episodes were tested for A(H1N1)pdm09 by qRT-PCR; acute and 60-day convalescent blood samples were tested by A(H1N1)pdm09 hemagglutination inhibition assay (HI). Enrollment, 12-month and 24-month follow-up blood samples were tested for A(H1N1)pdm09 seroconversion by HI. Household members of influenza A-infected cohort subjects with ILI were enrolled in household transmission investigations in which day 0 and 60 blood samples and acute respiratory samples were tested by either qRT-PCR or HI for A(H1N1)pdm09. Seroconversion between annual blood samples without A(H1N1)pdm09-positive ILI was considered as subclinical infection.

**Results:**

The 2-yr cumulative incidence of A(H1N1)pdm09 infection in the cohort in 2009/2010 was 10.8% (84/781) with an annual incidence of 1.2% in 2009 and 9.7% in 2010; 83.3% of infections were subclinical (50% in 2009 and 85.9% in 2010). The 2-yr cumulative incidence was lowest (5%) in adults born ≤1957. The A(H1N1)pdm09 secondary attack rate among household contacts was 47.2% (17/36); 47.1% of these infections were subclinical. The highest A(H1N1)pdm09 secondary attack rate among household contacts (70.6%, 12/17) occurred among children born between 1990 and 2003.

**Conclusion:**

Subclinical A(H1N1)pdm09 infections in Thai adults occurred frequently and accounted for a greater proportion of all A(H1N1)pdm09 infections than previously estimated. The role of subclinical infections in A(H1N1)pdm09 transmission has important implications in formulating strategies to predict and prevent the spread of A(H1N1)pdm09 and other influenza virus strains.

## Introduction

Pandemic influenza A(H1N1)pdm09 emerged in North America in March 2009 and rapidly spread to other parts of the world, eventually replacing previous seasonal H1N1 virus strains. Several studies from different countries established a pattern of higher A(H1N1)pdm09 infection rates in school-age children relative to young adults, with older adults having the lowest infection rates [1]–[6]. These studies were primarily community-based serological surveys that relied on non-prospective cohort designs and convenience sampling rather than sampling of prospective longitudinal cohorts. Household transmission studies were also conducted during the 2009 pandemic to characterize A(H1N1)pdm09 transmission in confined settings. Several household studies reported a wide range (8% to 45%) of secondary attack rates [7]–[10]. These studies were mainly conducted during the early stages of the pandemic (April to August 2009) based on household investigations usually triggered by sentinel A(H1N1)pdm09 cases. In addition, some studies reported subclinical A(H1N1)pdm09 infection rates of 9% to 50% of all A(H1N1)pdm09 infections in adults [1], [7], [11]–[13], and 9% to 25% among household members [7]–[10]. Estimating the proportion of subclinical infections is relevant because these infections may contribute to virus transmission [14].

**Figure 1:**
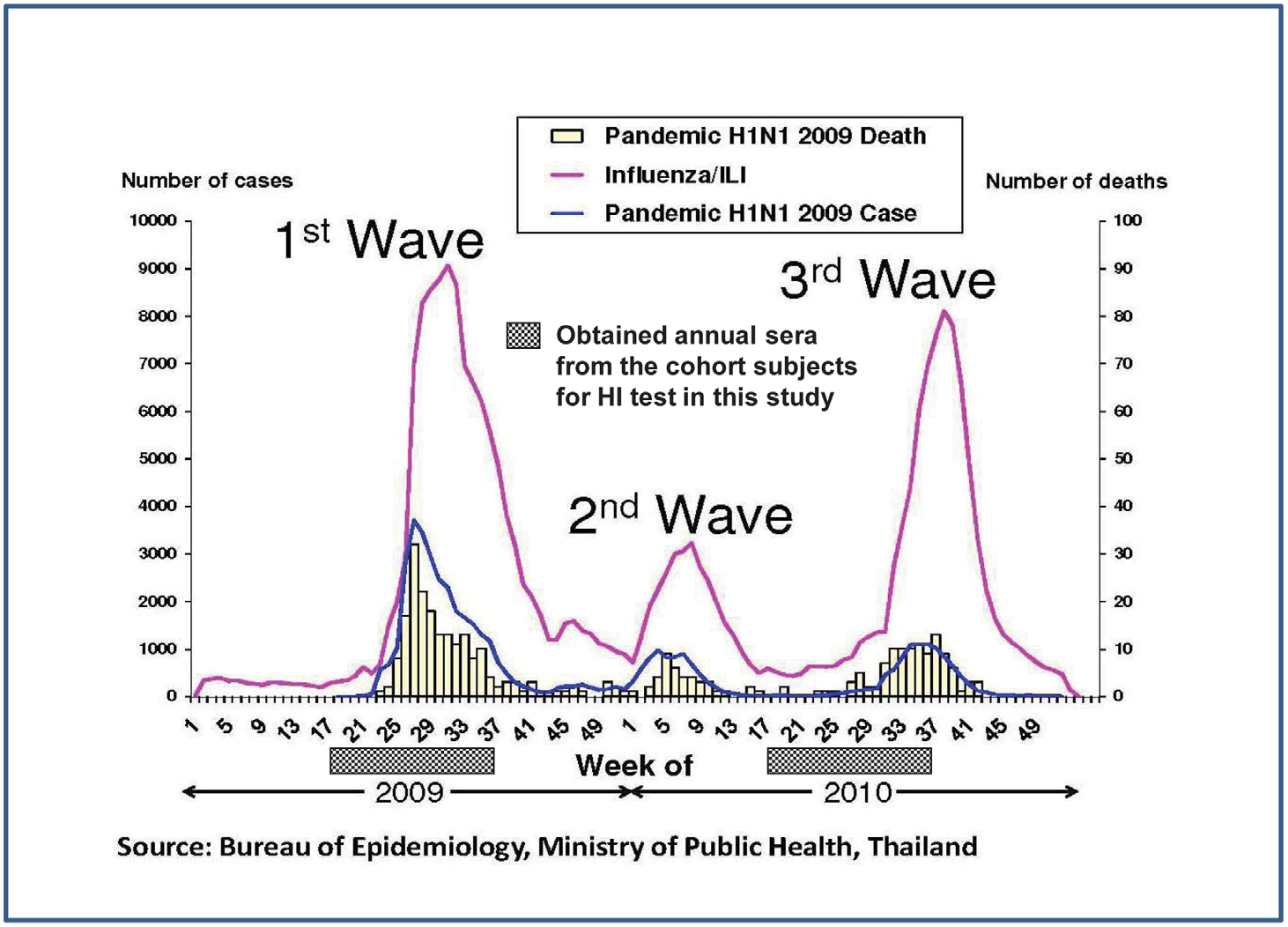
Three waves of the pandemic influenza A(H1N11)pdm09 in Thailand. Source: Bureau of Epidemiology, Ministry of Public Health, Thailand.

In Thailand, like other tropical countries, influenza activity tends to occur in a biphasic seasonal pattern (June to August and January to March) with sporadic infection throughout the year [15]. Between May 2009 and November 2010, three waves of the influenza pandemic influenza A(H1N1)pdm09 were clearly documented in Thailand (Figure 1). During this 18-month period, it was estimated that nearly one half of the Thai population may have been infected by the pandemic influenza A(H1N1)pdm09 [16].

At the time of pandemic onset in 2009, a prospective longitudinal cohort study was ongoing in Kamphaeng Phet, Thailand in which 800 adults (age range 20–85 years) were undergoing active surveillance for influenza-like illness (ILI) from 2008 to 2010. The study also included a household transmission component triggered by confirmed influenza virus (IFV) infections in the cohort [17]–[18]. This provided a unique opportunity to investigate the epidemiology and transmission of A(H1N1)pdm09, yielding information about A(H1N1)pdm09 infection rates in adults and their household members in a community setting, and providing estimates of subclinical infection rates and risk factors.

## Materials and Methods

### Ethics Statement

The study was approved by the institutional review boards of the Thai Ministry of Public Health (MOPH), Walter Reed Army Institute of Research (WRAIR), Naval Medical Research Unit No. 2 (NAMRU-2), US Army Medical Research and Materiel Command, University of Iowa, and University of Florida. Written informed consent was obtained from all study subjects (or their parents if applicable) and assent was obtained from children older than seven years.

Although the study was designed to focus chiefly upon studying subclinical avian influenza infections, reviewing IRBs approved the study to include all influenza A infections.

### Prospective Longitudinal Cohort

Approximately 800 adult subjects living in Kamphaeng Phet province (KPP), Thailand, a rural province located about 350 km north of Bangkok, were enrolled in a prospective longitudinal cohort study. Details of study methods have been previously published [17]–[18]. Briefly, cohort subjects were enrolled from April to October 2008. Clinical and demographic questionnaires were administered, blood samples were obtained, and digital thermometers were provided at enrollment. Active surveillance for influenza-like illness (ILI) was conducted through weekly home visits by study staff. Weekly questionnaires were administered to detect any changes in clinical status. Subjects were also encouraged to contact study staff if they developed any febrile episodes. If the subject met the ILI case definition (measured temperature ≥38°C with sore throat or cough for ≥4 hours), an acute blood sample and acute respiratory samples (one nasal and one throat swab) were collected. At day 60 after the acute visit, a convalescent blood sample was collected. Semi-quantitative real-time reverse transcriptase polymerase chain reaction (qRT-PCR) was performed on respiratory samples to detect influenza A or B. Serological testing of the paired acute/convalescent blood samples was performed by hemagglutination inhibition assay (HI) against IFV.

Annual follow-up visits at 12 and 24 months after enrollment were performed in 2009 and 2010 during which clinical questionnaires were administered and blood samples were obtained. The questionnaires assessed changes in demographic or health status during the preceding year. Serological testing of the enrollment and annual follow-up blood samples was conducted by influenza HI to monitor for changes in influenza antibody titers over time. An IFV infection was considered to be subclinical if IFV HI seroconversion occurred between annual blood samples without an IFV-positive ILI detected during the intervening surveillance period.

### Household Transmission Investigations

When a cohort subject developed an ILI in which an acute respiratory sample tested positive for influenza A by qRT-PCR, that cohort subject’s household members ≥6 months old were enrolled in a household transmission investigation. A blood sample was collected at enrollment (day 0) from each household member (i.e., contact subject). If a contact subject developed ILI during the following 60 days, acute respiratory samples (one nasal and one throat swab) were additionally collected from that contact subject for qRT-PCR for influenza A and B. A questionnaire was administered to each contact subject to assess the degree of contact with the cohort subject and identify any illnesses during the preceding 7 days. Weekly follow-up visits were conducted with the contact subjects for 60 days after initiation of the household investigation. At day 60, a convalescent blood sample was collected from each contact subject.

### Laboratory Methods

Blood samples were transported at room temperature and respiratory swabs were transported in Micro Test M4RT Viral Transport Media (Remel, Inc., Lenexa, KS, USA) at 4°C within the same day to the field laboratory at Kamphaeng Phet-AFRIMS Virology Research Unit (KAVRU) in KPP, Thailand, where qRT-PCR for IFV was performed. Serological testing by HI was performed at the University of Florida Global Pathogens Laboratory in Gainesville, Florida, USA.

#### Hemagglutination Inhibition (HI) assay

Enrollment and annual follow-up serum samples were tested using the World Health Organization (WHO) or US Centers for Disease Control and Prevention (CDC) HI protocol as previously described [17], [19] against 2 human viruses: A/Brisbane/59/2007(H1N1), and A/Mexico/4108/2009(H1N1); and 2 swine viruses: A/SW/Gent/7625/99(H1N2) and A/SW/Lutol/3/2000(H1N1). Acute and convalescent serum samples collected from ILI episodes were tested against A/Mexico/4108/2009(H1N1). IFV strains were grown in fertilized eggs. Sera were pretreated with receptor-destroying enzyme and hemabsorbed with either guinea pig or turkey erythrocytes. Titer results were reported as the reciprocal of the highest dilution of serum that inhibited virus-induced hemagglutination of 0.65% (guinea pig) or 0.50% (turkey) solution of erythrocytes as previously described [17]. An HI titer of ≥1∶40 was considered as positive. Seroconversion between paired samples was considered to be 4-fold or greater rise in HI titer.

#### Semi-quantitative real-time qRT-PCR

The procedures have been previously described [18]. Briefly, RNA was extracted from 140 µl of each nasal and throat swab using QIAamp viral RNA extraction kit (Qiagen Inc., Valencia, CA, USA) and screened via a qRT-PCR protocol developed by the US CDC. The protocol was designed to first screen for influenza A, and then through separate reactions, to rapidly determine influenza subtype. The human RNase P gene primer set was also used as an internal positive control for human RNA in each specimen.

Infection with A(H1N1)pdm09 was considered to have occurred if (a) a respiratory sample was positive for A(H1N1)pdm09 by qRT-PCR, or (b) a 4-fold or greater increase in HI titer (seroconversion) against A(H1N1)pdm09 occurred between paired annual sera or paired ILI sera, with a second titer of at least 1∶40. If paired sera were not available, a single high titer of at least 1∶40 was considered to indicate recent infection.

### Statistical Methods

Risk factors for bivariate associations with HI results were initially examined using binary logistic regression and proportional odds modeling [20]. An exact method was used for sparse data, and the score test was used to evaluate the proportional odds assumption. Covariates with p values <0.25 were considered for inclusion in multivariate models. Final multivariate models were designed using manual backwards elimination. Analyses were performed using SAS v9.2 (SAS Institute Inc., Cary, NC, USA).

## Results

Between April and October 2008, field staff enrolled a total of 800 adults (100 from each of 8 sites). Details of participant demographics at enrollment have been previously described [17]. The median age of the 800 participants was 49.6 years. Only 1.6% of the cohort reported ever receiving a human influenza vaccine and 11.4% reported ever being exposed to swine. Over the 24-month follow-up period, 49 subjects withdrew their participation and 45 replacement enrollments were added. A total of 768 participants (96%) completed the 12-month annual follow-up and 784 participants (98%) completed the 24-month annual follow-up visit. Overall, 747 participants (93%) remained enrolled for the entire study duration by completing enrollment and both 12- and 24-month follow-up visits.

### Prospective Longitudinal Cohort

Seroprevalence of elevated HI antibodies against A(H1N1)pdm09 increased from 1.7% and 1.3% in 2008 and 2009, respectively, to 10.3% in 2010 (Table 1). Based on serological results from 2009, cohort subjects born ≤1957 were more likely to have elevated A(H1N1)pdm09 antibodies compared to those born after 1957 (Table 1); however, in 2010, cohort subjects born after 1957 were more likely to have positive HI titer against A(H1N1)pdm09. The birth year of 1957 was chosen as the cut-point since it has been shown that immunity to A(H1N1)pdm09 virus could be acquired as a result of previous exposure to a 1918-like A(H1N1) virus circulating during the period 1918–1957 [1]. The seroconversion rate in those born after 1957 was ≥12%, more than double the rate in those born ≤1957 (Table 1). None of the subjects who seroconverted to A(H1N1)pdm09 were seropositive (i.e., A(H1N1)pdm09 HI titer ≥1∶40) in their 2009 sera.

**Table 1:** Evidence of A(H1N1)pdm09 infection among cohort subjects, Kamphaeng Phet, Thailand.

Category	Birth Year			Total (%)
	≤1957 (%)	1958–1968 (%)	≥1969 (%)	
**Seroprevalence** a				
(HI titer ≥1∶40)				
Enrollment (2008)	4/280 (1.4)	4/207 (1.9)	3/159 (1.9)	11/646 (1.7)
12-month (2009)	7/338 (2.1)	2/243 (0.8)	2/186 (1.1)	11/767 (1.4)
24-month (2010)	21/339 (6.2)	34/252 (13.5)	26/199 (13.1)	81/790 (10.3)
**Seroconversion** b	17/337 (5.0)	31/249 (12.4)	25/195 (12.8)	73/781 (9.3)
**A(H1N1)pdm09 infection** c	21 (2.7)	34 (4.4)	29 (3.7)	84 (10.8)
**Subclinical infection** d	15 (17.9)	31 (36.9)	24 (28.6)	70 (83.3)

a HI testing not conducted in some subjects due to insufficient serum volume.

b ≥4-fold increase in HI titer of 2009/2010 sera.

c Positive respiratory sample for A(H1N1)pdm09 by qRT-PCR or ≥4-fold increase in HI titer of paired annual sera/paired ILI sera.

d ≥4-fold increase in HI titer of paired annual sera/paired ILI sera without positive respiratory sample for A(H1N1)pdm09 by qRT-PCR.

From January 2009 to December 2010, 53 cohort subjects met the case definition for ILI; 10 of these subjects were confirmed to have A(H1N1)pdm09 infection by qRT-PCR. Most (70%, 7/10) of these subjects were born after 1957. Prior to ILI occurrence, none of the 10 subjects had pre-existing A(H1N1)pdm09 antibody. Only 3 of these subjects had A(H1N1)pdm09 seroconversion in the paired ILI sera. Among the remaining 7 subjects with A(H1N1)pdm09-positive qRT-PCR, 5 were seronegative in both of the paired ILI sera and 2 did not have sufficient paired sera for HI testing.

When comparing A(H1N1)pdm09 seroconversions from the enrollment and annual follow-up blood samples and the qRT-PCR data from ILI acute respiratory samples, 5 subjects had A(H1N1)pdm09 infection by both qRT-PCR and HI, 5 by qRT-PCR only, and 74 by HI alone. Among these 84 cohort subjects with A(H1N1)pdm09 infection, only 14 subjects reported ILI [10 were A(H1N1)pdm09-positive by qRT-PCR; 4 were negative by qRT-PCR]. Most (83.3%, 70/84) of the A(H1N1)pdm09 infections among the cohort subjects were subclinical. The proportion of subclinical infections among cohort subjects born after 1957 was higher than those born ≤1957 (Table 1).

Using binary logistic regression analysis, several covariates were associated with an increased risk for seroconversion against A/Mexico/4108/2009(H1N1) (Table 2). Cohort subjects born after 1957 were more likely to have seroconversion than those born ≤1957 (unadjusted OR = 2.7, 95% CI = 1.4–5.0 for subjects born between 1958 and 1968; unadjusted OR = 2.8, 95% CI = 1.5–5.3 for subjects born after 1968). Indoor water use was associated with an increased risk for seroconversion (unadjusted OR = 2.1, 95% CI = 1.2–3.9). Cohort subjects living in Phran Kratai district were more likely to have seroconversion than those living in Mueng district (unadjusted OR = 2.7, 95% CI = 1.7–4.4). Elevated titers against A/SW/Gent/7625/99(H1N2) and A/SW/Lutol/3/2000(H1N1) were also associated with A(H1N1)pdm09 seroconversion (unadjusted OR = 6.4, 95% CI = 3.8–10.7; unadjusted OR = 8.2, 95% CI = 3.6–18.6, respectively). There was a trend for elevated antibody against seasonal A/Brisbane/59/2007(H1N1) to be associated with an increased risk of seroconversion against pandemic A/Mexico/4108/2009(H1N1) (unadjusted OR = 1.7, 95% CI = 0.9–2.9).

**Table 2:** Risk factors for ≥4-fold increase in hemagglutination inhibition (HI) titer against A/Mexico/4108/2009(H1N1) from 2009 to 2010 among cohort subjects, Kamphaeng Phet, Thailand; odds ratios calculated by binary logistic regression.

Variables	Total N	A/Mexico/4108/2009(H1N1)		
		≥4-fold	Unadjusted OR (95% CI)	Adjusted OR (95% CI)
		increase		
		N (%)		
Birth Year				
>1968	195	25 (34.2)	2.8 (1.5–5.3)	2.2 (1.1–4.3)
1958–1968	249	31 (42.5)	2.7 (1.4–5)	2.5 (1.3–4.8)
≤1957	337	17 (23.3)		Reference
Gender				
Male	318	29 (39.7)	1 (0.6–1.6)	-
Female	463	44 (60.3)		
Indoor Water				
Yes	528	59 (80.8)	2.1 (1.2–3.9)	-
No	253	14 (19.2)		
Geographical Region				
Phran Kratai District	298	44 (60.3)	2.7 (1.7–4.4)	2.6 (1.5–4.5)
Mueng District	483	29 (39.7)		Reference
Swine Exposure				
Yes	87	7 (9.6)	0.8 (0.4–1.9)	-
No	694	66 (90.4)		
Developed a respiratory illness in the last 12 months^a^				
Yes	423	44 (60.3)	1.3 (0.8–2.2)	-
No	357	29 (39.7)		
A/Brisbane/59/2007(H1N1)				
Positive	143	19 (26)	1.7 (1.0–2.9)	-
Negative	638	54 (74)		
A/SW/Gent/7625/99(H1N2)				
Positive	211	48 (65.8)	6.4 (3.8–10.7)	5.8 (3.4–10.0)
Negative	570	25 (34.2)		Reference
A/SW/Lutol/3/2000(H1N1)				
Positive	26	11 (15.1)	8.2 (3.6–18.6)	3.7 (1.5–9.6)
Negative	755	62 (84.9)		Reference

Abbreviation: OR, odds ratio; CI, confidence interval.

a Covariate has some missing values.

### Household Transmission Investigations

Thirty-six contact subjects (age range 1–70 years) living in the same household as the 10 cohort subjects with A(H1N1)pdm09-positive qRT-PCR were enrolled. Paired day 0 and 60 sera from these contact subjects were evaluated by HI against A(H1N1)pdm09 [A/Mexico/4108/2009(H1N1)]; three contact subjects missed their day 60 blood draw. Of the 36 contact subjects, 6 had ILI of which all were A(H1N1)pdm09-positive by qRT-PCR (Table 3). Five of these 6 contact subjects had HI seroconversion against A(H1N1)pdm09, and one was seronegative at both day 0 and 60. Of the 30 contact subjects without ILI, 8 had seroconversion against A(H1N1)pdm09, and 3 had elevated antibody against A(H1N1)pdm09. Of the 8 contact subjects with seroconversion, 3 reported having a respiratory illness within 7 days prior to enrollment. None of the 3 seropositive contact subjects reported having a respiratory illness within 7 days prior to enrollment. Therefore, the A(H1N1)pdm09 infection rate among household members was 47.2% (17/36) and the proportion with subclinical infection was 47.1% (8/17) (Table 4). The majority (88.2%, 15/17) of A(H1N1)pdm09 infections among contact subjects was in those individuals born after 1957. The highest infection rate (70.6%, 12/17) was in school-age children (born between 1990 and 2003) as shown in Table 4.

**Table 3:** Description of contact subjects living with A(H1N1)pdm09-infected cohort subjects, Kamphaeng Phet, Thailand.

Year ofInfection	ID of Cohort Subject withA(H1N1)pdm09 Infection	Number of EnrolledContacts/Total HouseholdMembers	Number of Contact Subjects with:				
			ILI	A(H1N1)pdm09–positiveby qRT-PCR	HemagglutinationInhibition (HI)		
					Titer against		
					A/Mexico/4108/2009(H1N1)		
					Seroconversion^a^	Positive^b^	Negative
**2009**	T20133AIS	6^c^/6	1	1	0	0	6
	T50492AIS	1/6	0	0	0	1	0
	T20159AIS	9/9	5	5	6	0	3
**2010**	T70655AIS	3/3	0	0	0	1	2
	T40378AIS	2/2	0	0	2	0	0
	T40379AIS	2^c^/2	0	0	0	0	2
	T50448AIS	6/6	0	0	3	0	3
	T80750AIS	2/2	0	0	1	1	0
	T20119AIS	2/2	0	0	1	0	1
	T20129AIS	3^c^/5	0	0	0	0	3
**Total**	**N = 10**	**36/43**	**6**	**6**	**13**	**3**	**20**

a ≥4-fold increase in HI titer from day 0 to day 60 sera.

b Single positive HI titer ≥1∶40.

c Missing convalescent sample for one subject.

**Table 4:** Age distribution of contact subjects with laboratory-confirmed A(H1N1)pdm09 infection, Kamphaeng Phet, Thailand.

Birth Year	Number ofEnrolledContactSubjects	Number ofA(H1N1)pdm09-positive byqRT-PCR^a^	Number ofA(H1N1)pdm09-positive by Seroconversionor Single Positive HI Titer	Number of Symptomatic^b^A(H1N1)pdm09 Infection	Number of Subclinical^c^A(H1N1)pdm09 Infection
≤1957	5	0	2	0	2
1958–1968	2	1	2	2	0
1969–1989	9	1	1	1	0
1990–2003	16	3 (1)	9	4	6
≥2004	4	1	2	2	0
**Total**	**36**	**6 (1)**	**16**	**9**	**8**

a Number in parenthesis refers to sample determined to be positive by qRT-PCR without documented seroconversion or single positive HI titer.

b Symptomatic infections include those having positive respiratory sample for A(H1N1)pdm09 by qRT-PCR or ≥4-fold increase in HI titer of paired ILI sera or single positive HI titer with ILI or respiratory illness within 7 days prior to study enrollment.

c Subclinical infections include those having ≥4-fold increase in HI titer of paired ILI sera or single positive HI titer without ILI or respiratory illness within 7 days prior to study enrollment.

## Discussion

Our report is one of the few combined prospective longitudinal cohort and household transmission studies conducted during the A(H1N1)pdm09 pandemic that describes the epidemiology of A(H1N1)pdm09 in a tropical region. The proportion of A(H1N1)pdm09 infections in our cohort that were subclinical was very high (83.3%). This proportion was higher than some prior estimates (approximately 50%) from a few comparable studies [11]–[13]. This may be due to differences in our study design. For example, the age range of our cohort population was between 20–85 years (mean age = 49.6 years), whereas the age range in the study by Aho et al. [11] and Khaokham et al. [13] was 20–28 years (mean age = 21 years) and 19–58 years (median age = 24.7 years), respectively. In addition, the proportion of adults older than 60 years in our study (23%) was greater than in the community cohort study by Chen et al. [12] (7%). Our age composition was, however, unlikely to be the only factor in these differences since the rate of subclinical infections in our cohort was high even among those born after 1957. An additional possibility is that active surveillance in our cohort was conducted before and during the course of the pandemic from April 2008 to December 2010. Aho et al. [11], Chen et al. [12], and Khaokham et al. [13] conducted their studies during the early stages of the pandemic (i.e., November 2009, June to October 2009, and September to October 2009, respectively). The rate of subclinical A(H1N1)pdm09 infection in the current study is consistent with that from a recently published multi-year prospective household-based cohort study in Vietnam conducted from 2007 to 2010 [21]. That study reported 84.4% of A(H1N1)pdm09-infected subjects did not have ILI. Subclinical A(H1N1)pdm09 infection may contribute to a substantial fraction of virus transmission as suggested by a simulation study [14]. The household transmission investigations in our study also revealed a high subclinical A(H1N1)pdm09 infection rate among household contacts (47.1%) which was higher than the range (9% to 25%) reported by previous household transmission studies conducted during the early pandemic phase from April to August 2009 [7]–[10]. To confirm infections among household contacts, Suess et al. [10] used only qRT-PCR, while Jackson et al. [8] performed only serological testing. On the other hand, Cowling et al. [7] and Papenburg et al. [9] included both qRT-PCR and serological testing for A(H1N1)pdm09 infection.

Cohort subjects born ≤1957 (age ≥52 years) were less likely to have A(H1N1)pdm09 seroconversion, comparable to the findings from other studies [12]. Multivariate analyses suggested that adults born after 1957 were more susceptible to A(H1N1)pdm09 infection (adjusted OR = 2.1, 95% CI, 1.1–4.2 for subjects born after 1968; adjusted OR = 2.4, 95% CI, 1.2–4.6 for those born between 1958 and 1968). These findings support the hypothesis that older adults may have acquired partial immunity to A(H1N1)pdm09 from previous exposure to a 1918-like A(H1N1) virus circulating between 1918 and 1957 [1], or from a lifetime of exposure to influenza A resulting in broad heterotypic immunity [22]–[26]. Cross-reactive antibody to the A(H1N1)pdm09 virus has also been demonstrated in archival serum samples from adult recipients of trivalent inactivated influenza vaccines during 2007–2009 seasons [23]. However, only 4 of 84 cohort subjects with A(H1N1)pdm09 infection reported ever receiving human influenza vaccines. Hence, we posit that a high prevalence of cross-reacting antibody to the A(H1N1)pdm09 virus seems unlikely. Local geographical area was also associated with A(H1N1)pdm09 infections. Subjects living in Phran Kratai district had higher adjusted odd ratios for A(H1N1)pdm09 infection (adjust OR = 2.6; 95% CI, 1.5–4.5) compared with those living in Mueng district. Since Phran Kratai district has approximately 3 times more pigs per person than does Mueng district (KPP Livestock Office data), cohort subjects living in Phran Kratai district may have had antibodies to swine viruses which were cross-reactive with A(H1N1)pdm09. This is supported by the fact that elevated antibodies against the two swine viruses, A/SW/Gent/7625(H1N2) and A/SW/Lutol3/2000(H1N1), were associated with A(H1N1)pdm09 seroconversion among the cohort subjects (Table 2). A similar finding was reported by Kyriakis et al. [27] that pigs infected or vaccinated with European swine influenza viruses had cross-reactive antibodies against A(H1N1)pdm09. It is of note that swine exposure was not associated with the A(H1N1)pdm09 infection (Table 2).

The cumulative incidence of A(H1N1)pdm09 infection by qRT-PCR and HI seroconversion in the cohort from January 2009 to December 2010 was 10.8% with an annual incidence of 1.2% and 9.7% in 2009 and 2010, respectively. Previous studies estimated the incidence of A(H1N1)pdm09 infection among adults to range from 6% to 13% during the early pandemic (May to September 2009) [2]–[4], [6], [12] and from 3% to 41% during November 2009 to May 2010 [28]–[31]. These wide ranges are likely a result of varying study designs, study periods, laboratory methods, and sample sources.

The A(H1N1)pdm09 secondary attack rate (SAR) among household members of cohort subjects was 47.2% (50% in 2009, 45% in 2010). Previous A(H1N1)pdm09 household transmission studies conducted during the early pandemic (April to August 2009) reported a wide range (4% to 45%) of SARs [8]–[10], [32]–[35]. Most of these studies used only clinical symptoms (ILI) [32]–[33] or serology [8] to identify secondary A(H1N1)pdm09 cases, or performed qRT-PCR only for clinically suspected secondary cases [10], [34]–[35]. In contrast, the study by Papenburg et al. [9] sought secondary A(H1N1)pdm09 cases among all consented household members (symptomatic or not) performing laboratory diagnostic testing to include serology and qRT-PCR. That study reported a SAR of 45% for laboratory-confirmed A(H1N1)pdm09 similar to the SAR in our study. It is well accepted that influenza can present with a broad range of symptoms and severities, including atypical and subclinical infections. Thus, underestimation of IFV infection by 25–50% may occur depending on the surveillance system and diagnostic criteria [36]–[40]. As demonstrated in previous studies [9], [41] and substantiated by our work, serological evaluation in combination with molecular analysis (qRT-PCR) can improve the sensitivity of case detection.

Our study had some limitations. Only adults over 20 years of age were enrolled in the cohort. Since children are at high risk of A(H1N1)pdm09 infection [42], our sampling method may have excluded a substantial portion of individuals at-risk. However, children were indeed included in the household transmission investigations in which school-aged subjects were found to have the highest A(H1N1)pdm09 infection rate. The small sample size in the household transmission component of our study limited what conclusions could be made. Nevertheless, the A(H1N1)pdm09 secondary attack rate among household members in our study was similar to those demonstrated in previous studies. Other limitations are related to the serological assay. It is possible that HI seropositivity against A(H1N1)pdm09 may have been due to cross-reactive IFV strains. Furthermore, the HI cut-off of 1∶40 was chosen in order to be comparable with other studies; however, different cut-offs would change our findings.

Our study was unique in evaluating the 2009 influenza pandemic in several ways. First, it combined a prospective longitudinal cohort study undergoing active ILI surveillance with a household transmission component. Second, it was already fully operational at the outset of the pandemic. Third, it continued operating for the full duration of the pandemic. Keeping these aspects of the study in mind, our results demonstrate that subclinical A(H1N1)pdm09 infection occurred frequently among Thai adults in this rural population. The role of these subclinical infections in A(H1N1)pdm09 transmission has important implications in formulating strategies to predict and control the spread of A(H1N1)pdm09 and other IFV strains. For instance, if a large portion of viral infections are subclinical, hospital-based surveillance will not well-represent a population’s influenza ecology. Hence, public health officials should consider these large numbers of subclinical infections in developing future influenza surveillance and control programs. The effort to capture subclinical data need not be as extensive and expensive as this cohort study. For example, subclinical infection data might be collected through modest household transmission studies, where secondary attack rates (clinical and subclinical) among persons in the same household as index cases may be used as a surrogate for general population data.
